# Complete Genome Sequence of “*Candidatus* Hydrogeosomobacter endosymbioticus,” an Intracellular Bacterial Symbiont of the Anaerobic Ciliate Scuticociliate GW7

**DOI:** 10.1128/mra.01150-21

**Published:** 2022-02-17

**Authors:** Yasuo Shiohama, Kazutaka Takeshita, Yuga Hirakata, Masaru K. Nobu, Michihiro Ito, Naoya Shinzato

**Affiliations:** a Tropical Biosphere Research Center, University of the Ryukyus, Nishihara, Okinawa, Japan; b Faculty of Bioresource Sciences, Akita Prefectural University, Akita, Japan; c Bioproduction Research Institute, National Institute of Advanced Industrial Science and Technology (AIST), Tsukuba, Japan; Indiana University, Bloomington

## Abstract

The bacterium “*Candidatus* Hydrogenosomobacter endosymbioticus” is an intracellular symbiont of anaerobic scuticociliate GW7, which is associated with hydrogenosome together with methanogenic archaea. Here, we report a complete genome sequence of the symbiont consisting of 827 kbp. Knowing this sequence would contribute to the understanding of the metabolic interactions and evolution of the tripartite symbiosis.

## ANNOUNCEMENT

The free-living anaerobic ciliate scuticociliate GW7 isolated from sewage treatment tank harbors a symbiotic bacterium named “*Candidatus* Hydrogenosomobacter endosymbioticus,” a *Holospora*-related alphaproteobacterium (1). The symbiotic bacterium was closely associated with a hydrogen-producing organelle, hydrogenosome, together with *Methanoregula*-related methanogenic archaeal symbiont (2, 3). In this announcement, we attempted to determine the whole genome sequence of “*Ca*. Hydrogenosomobacter endosymbioticus” by metagenomic approach.

The cells of “*Ca*. Hydrogenosomobacter endosymbioticus” were fractionated from a total of 4 L of ciliate cultures as described previously (1, 4–6). After starvation for 18 h in fresh medium, the ciliate cells were homogenized with a tissue grinder. The symbiont cells were fractionated by passing through a 5-μm membrane filter (Millipore) to remove ciliate cell debris and subjected to DNA extraction using REDExtract-N-Amp tissue PCR kit (Sigma-Aldrich). Finally, 25 ng of DNA was used for transposome-mediated library preparation using Nextera DNA Flex library prep kit (Illumina). The library was sequenced using MiSeq sequencer (Illumina) with MiSeq reagent kit v3 (Illumina).

The obtained reads were assembled as described below with default parameters except where otherwise noted. The obtained reads were trimmed by 33 of phred score and the reads less than 35 bp were discarded using Trimmomatic v0.36 (7). A total of 28,874,984 reads were assembled using SPAdes v3.13.0 (8). The obtained contigs were clustered by binning using CONCOCT v1.1 (9), Maxbin2 v2.2.4 (10), and MetaBAT2 v1.7 (11). Finally, 22 consensus bins were classified using GTDB-Tk classify v1.1.0 (12). Based on the results, we determined that one of the bins was the genome of “*Ca*. Hydrogenosomobacter endosymbioticus.” This genome bin contained 88 contigs, and an *N*_50_ was 825,562 bp. To examine the circularity of the longest contig (825,562 bp), the symbiont genome was amplified by PCR with a set of primers, 5′-CCTGCTCTGGCGTTAATTGTTGTG-3′ and 5′-CCGCTCTTCTTCCGTCAGCCTTCCATAG-3′, which were designed for each end of the longest contig. The PCR product (15,367 bp) was inserted into pCR4-TOPO vector (Thermo Fisher Scientific) and sequenced by primer walking. The obtained sequences were aligned to the longest contig, and finally, a circular chromosome of 826,669 bp was obtained. The genome completeness estimated by CheckM was 89.6%, which is a program for assessing the genome completeness based on the retention rate of marker genes (13). The sequencing depth estimated by mapping reads to circular genome was 81×. The open reading frame (ORF) detection and gene annotation have been performed using DFAST pipeline v1.2.13 (14, 15). As the results, 721 protein-coding sequences, 45 tRNAs, and 3 rRNAs were detected. The GC content was 41.3%.

The reported genome sequence will make clear the physiological features of “*Ca*. Hydrogenosomobacter endosymbioticus” and help deepen understanding of the microbial symbiotic association in anaerobic environments.

### Data availability.

The genome sequence of “*Ca*. Hydrogenosomobacter endosymbioticus” has been deposited in the DNA Data Bank of Japan (DDBJ) under accession number AP025225. The associated BioProject, BioSample, and SRA accession numbers are PRJDB12495, SAMD00412742, and DRA013104, respectively.
